# Estimating Relative Abundance of 2 SARS-CoV-2 Variants through Wastewater Surveillance at 2 Large Metropolitan Sites, United States

**DOI:** 10.3201/eid2805.212488

**Published:** 2022-05

**Authors:** Alexander T. Yu, Bridgette Hughes, Marlene K. Wolfe, Tomas Leon, Dorothea Duong, Angela Rabe, Lauren C. Kennedy, Sindhu Ravuri, Bradley J. White, Krista R. Wigginton, Alexandria B. Boehm, Duc J. Vugia

**Affiliations:** California Department of Public Health, Richmond, California, USA (A.T. Yu, T. Leon, A. Rabe, S. Ravuri, D.J. Vugia);; California Department of Public Health, Sacramento, California, USA (A.T. Yu, T. Leon, A. Rabe, S. Ravuri, D.J. Vugia);; Verily Life Sciences, South San Francisco, California, USA (B. Hughes, D. Duong, B.J. White);; Stanford University, Stanford, California, USA (M.K. Wolfe, L.C. Kennedy, A.B. Boehm);; Emory University, Atlanta, Georgia, USA (M.K. Wolfe);; University of Michigan, Ann Arbor, Michigan, USA (K.R. Wigginton)

**Keywords:** COVID-19, coronavirus disease, SARS-CoV-2, severe acute respiratory syndrome coronavirus 2, viruses, respiratory infections, zoonoses, vaccine-preventable diseases, wastewater, public health, environmental monitoring, United States

## Abstract

Monitoring severe acute respiratory syndrome coronavirus 2 (SARS-CoV-2) variants of concern (VOCs) is critical for public health management of coronavirus disease. Sequencing is resource-intensive and incompletely representative, and not all isolates can be sequenced. Because wastewater SARS-CoV-2 RNA concentrations correlate with coronavirus disease incidence in sewersheds, tracking VOCs through wastewater is appealing. We developed digital reverse transcription PCRs to monitor abundance of select mutations in Alpha and Delta VOCs in wastewater settled solids, applied these to July 2020–August 2021 samples from 2 large US metropolitan sewersheds, and compared results to estimates of VOC abundance from case isolate sequencing. Wastewater measurements tracked closely with case isolate estimates (Alpha, r_p_ 0.82–0.88; Delta, r_p_ 0.97). Mutations were detected in wastewater even at levels <5% of total SARS-CoV-2 RNA and in samples available 1–3 weeks before case isolate results. Wastewater variant monitoring should be strategically deployed to complement case isolate sequencing.

By November 2021, the coronavirus disease (COVID-19) pandemic, caused by severe acute respiratory syndrome coronavirus 2 (SARS-CoV-2), had claimed >5 million lives worldwide, including >700,000 in the United States (*1*–*3*). Since its emergence in late 2019, SARS-CoV-2 has mutated, resulting in some variants categorized by the World Health Organization as variants of concern (VOCs). VOCs have evidence of potential increased infectiousness, immune evasion, and clinical severity, and they have spread globally. Some VOCs, such as Alpha and Delta, have become the predominant strain at different times and regions (*4*,*5*). COVID-19 diagnostics, therapeutics, or vaccines may have decreased effectiveness against VOCs (*6*,*7*). As of November 2021, VOCs in the United States included the Alpha (B.1.1.7), Beta (B.1.351), Gamma (P.1), and Delta (B.1.617.2) variants (*3*).

Monitoring for VOCs is critical for management of the ongoing COVID-19 pandemic, enabling public health officials to track their public health impact, implement control measures, and allocate resources effectively. Detection of SARS-CoV-2 variants occurs primarily through genomic sequencing of isolates collected for PCR-based diagnosis of persons with active COVID-19 infection. Sequencing is resource- and time-intensive and has limits on capacity because of equipment, reagents, and trained personnel (*8*). As such, complete and timely sequencing of case isolates is not feasible or practical, particularly when case numbers have been high. During January 2020–September 2021, <3% of COVID-19 cases in the United States had isolates that were sequenced and available on public repositories (*3*). Nonrandom selection of isolates for sequencing and nonuniform result reporting could make results susceptible to bias and not truly representative of circulating variants (*4*,*8*,*9*). Also, substantial delays can occur between isolate collection, sequencing and availability of results to public health (*9*). Given its timeliness, representativeness, and comparatively low costs, wastewater surveillance for VOCs can be a useful supplement to case-based sequencing surveillance (*10*–*12*).

Since early in the pandemic, wastewater samples have been collected and analyzed to quantify the amount of SARS-CoV-2 RNA in sewage. Estimates of viral RNA abundance in sewage correlate closely with reported COVID-19 case counts for the catchment area (sewershed) (*13*,*14*) and provide a comprehensive snapshot of real-time community transmission independent of individual care-seeking or testing behavior. Therefore, there is a strong interest in determining if wastewater can also provide useful information on circulating VOCs (*15*). Both sequencing and PCR assays targeting specific mutations have been proposed as methods to detect mutations and deletions in SARS-CoV-2 RNA in wastewater.

Variant monitoring using environmental samples presents technical challenges. Variants are characterized by the presence of multiple mutations on the same RNA genome, and some share >1 mutations (*16*). Unlike isolates from an individual case, which consist of a single genome, wastewater samples likely contain material from multiple SARS-CoV-2 variants shed from different persons, each variant at low concentrations and in various states of genomic integrity because of degradation (*17*). Therefore, because wastewater contains a complex mixture of SARS-CoV-2 RNA fragments, the presence of >1 variant mutation sequences does not alone prove that the variant is present in wastewater.

We developed targeted digital reverse transcription PCR mutation assays to retrospectively and prospectively monitor wastewater settled solids for the presence and abundance of mutations present in the Alpha (B.1.1.7) and Delta (B.1.617.2) VOCs. We chose wastewater solids because they contain orders of magnitude higher concentrations of viral RNA than wastewater influent (*18*,*19*); previous work has documented a strong coupling between SARS-CoV-2 RNA concentrations in wastewater solids and incidence in the associated population contributing to the wastewater (*19*). We prospectively monitored wastewater solids of a large metropolitan sewershed in California (San Jose), USA, during July 2020–August 2021 for a deletion present in the Alpha variant. We then retrospectively measured the abundance of this deletion in a second large metropolitan area (Sacramento, CA, USA) where samples had been routinely collected. We also measured concentrations of mutations suggestive of Delta in both sewersheds. We then compared these totals against estimates of Alpha and Delta abundance in each of these sewersheds by using COVID-19 case isolate sequencing data available to the California Department of Public Health (CDPH).

## Methods

### Mutation Assay Development for Alpha and Delta Variants

We developed assays in silico to target mutations present in Alpha (HV69-70) and Delta (Del156–157/R158G). We screened primers and probe sequences (Appendix Table) for specificity using BLAST (https://blast.ncbi.nlm.nih.gov/Blast.cgi), and then tested them in vitro against a wide range of viral genomes, including wild-type SARS-CoV-2 and SARS-CoV-2 VOCs, including Alpha and Delta. We further tested the sensitivity and specificity of the assays by diluting variant gRNA containing the mutations in no (0 copies), low (100 copies), and high (10,000 copies) background of wild-type gRNA (Appendix).

### Wastewater Sample Collection

This study used samples from 2 publicly owned treatment works (POTWs) that serve ≈1.5 million residents of Santa Clara County, California, USA (San Jose), and Sacramento County, California, USA (Sacramento). Details of collection processes have been described (*14*).

We collected samples from the POTWs to span the period before and including the presumed emergence of Alpha and Delta variants in the communities. Before presumed emergence, sampling was 1–4 times per month; during the periods of suspected emergence, sampling was 3–7 times per week. At San Jose, 133 (HV69–70) and 48 (del156–157/R158G ) samples and at Sacramento, 64 (HV69–70) and 48 (del156–157/R158G) samples were included for analyses of each mutation. 

We extracted RNA from the settled solids and processed within 24 hours of sample collection to measure concentrations of the nucleoprotein (N) gene using digital droplet reverse transcription PCR (Appendix) (*20*). The N gene codes for the SARS-CoV-2 nucleocapsid; the specific region of the genome targeted by the assay is conserved on SARS-CoV-2 genomes. We included internal recovery controls. Thereafter, we stored RNA samples at –80°C for 0–300 days before analyzing them a second time for the N gene and the Delta mutation (Del156–157/R158G) or the Alpha mutation (HV69-70), using digital droplet reverse transcription PCR. By comparing the N gene concentration in the samples before and after storage, we confirmed negligible RNA degradation. All wastewater data are publicly available (https://doi.org/10.25740/zf117dn1545).

### Incident COVID-19 Cases and Case Isolate Sequences

Each POTW provided sewershed boundary shapefiles. We determined the number of PCR-confirmed COVID-19 cases reported to CDPH as a function of episode date (earliest of either specimen collection or symptom onset date) residing within each sewershed using methods reported previously (*20*) (Appendix).

COVID-19 case isolate whole-genome sequence data available to CDPH included data from the CDC and laboratory partners. We assigned sequence data to a sewershed on the basis of residential home postal code for the sample. We assigned the PANGO lineage based on the software version available at the time data was extracted; most recent results used pangoLEARN and pango-designation version 1.2.66 (*21*).

We calculated VOC abundance estimates by dividing the number of sequences identified as Alpha or Delta (using the World Health Organization definition and including all PANGO sublineages Q.*, for Alpha, and AY.*, for Delta) by the total number of isolates sequenced from persons residing in the sewersheds over 14-day periods. To estimate time between isolate sample collection and sequence result and to measure the effect of that time delay on VOC estimates, we compared 14-day VOC abundance estimates over time against a final estimate generated on August 24, 2021. We chose a 14-day VOC window (versus a 7- or 28-day window) to balance timeliness of results and number of available case isolates sequenced within the window, given that fewer case isolates increase the uncertainty of estimates.

We performed Pearson correlations between the wastewater mutation and case isolate variant datasets, comparing the mean ratio of mutations in wastewater (HV69-70 and Del156-157/R158G to the N gene) to the proportion of case isolates sequenced and characterized as Alpha or Delta, each averaged over the previous 14 days. We used 0 as a replacement for samples where the measurement was below the limit of detection (nondetect); we repeated the analysis by using half the detection limit (500 copies/g), and the results were the same. We set statistical significance at p<0.05 and performed analyses in R studio version 1.4.1106 (https://www.rstudio.com).

## Results

### Variant Mutation Assay Specificity and Sensitivity

In silico analysis indicated no cross-reactivity between the assays and deposited sequences in GenBank. When challenged against wild-type gRNA, the respiratory virus panel, and actual or synthetic variant gRNA, no cross-reactivity occurred. Positive controls and no-template controls run on the sample plate performed according to expectations. Variant mutation concentrations were measured in no, low, and high background of wild-type gRNA, which does not contain the mutations. Results of mutation assays in the presence of high and low background wild-type gRNA are similar to their results in the absence of background wild-type gRNA (Appendix Figure 1), indicating that the assays are sensitive and specific. 

### Variant Mutation Concentrations in Wastewater Solids

**Figure 1:**
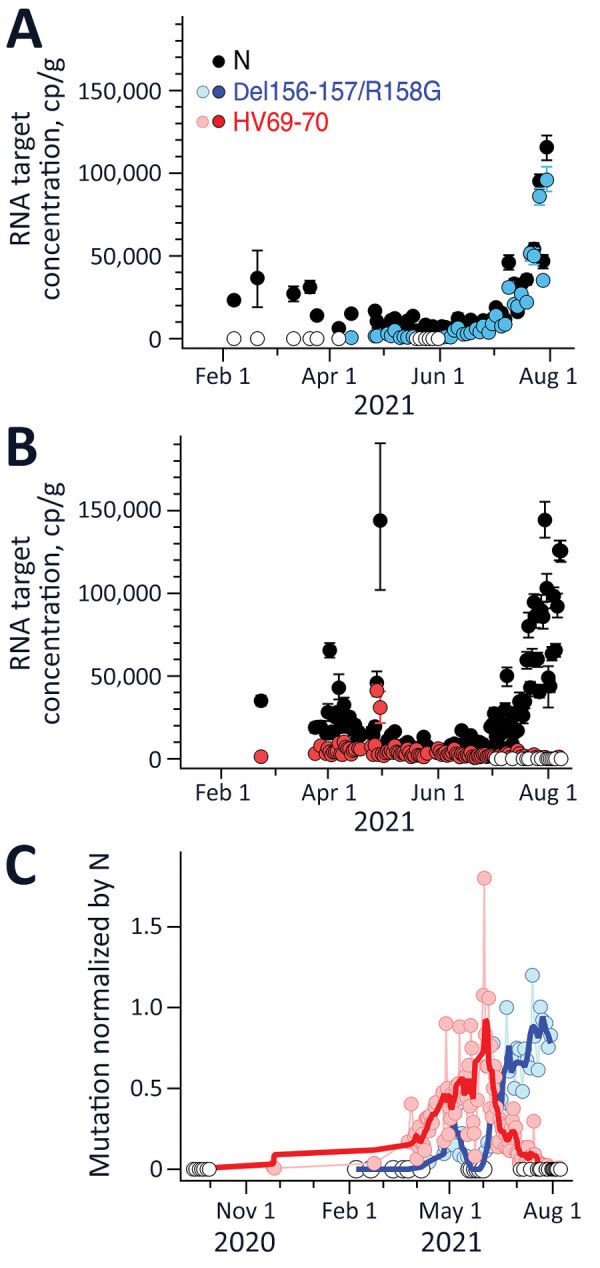
Measurements of severe acute respiratory syndrome coronavirus 2 variants of concern in wastewater solids, San Jose, California, USA. Concentrations of N gene and mutations found in Delta (Del156-157/R158G; panel A), and Alpha (HV69-70; panel B) variants in wastewater solids and their ratio (panel C). Error bars in panels A and B represent SDs derived from the 10 replicates run for each sample; open white circles are nondetects (below the limit of detection) and shown as 0. Errors include technical and replication errors. If error bars are not visible, then errors are smaller than the symbol. Panel C shows smoothed lines for visual reference for mutation ratios. For Del156-157/R158G/N ratio, the smoothed line is a 3-point running average, and for the HV69-70/N ratio, the smoothed line is a 7-point running average; each approximates a weekly average. The timescale for the HV69-70 data (B) is truncated for visualization; additional data on dates before February 15, 2021, are described in the article and shown (C), with the exception of data from July 14, 2020, which was nondetect. N, nucleoprotein.

**Figure 2:**
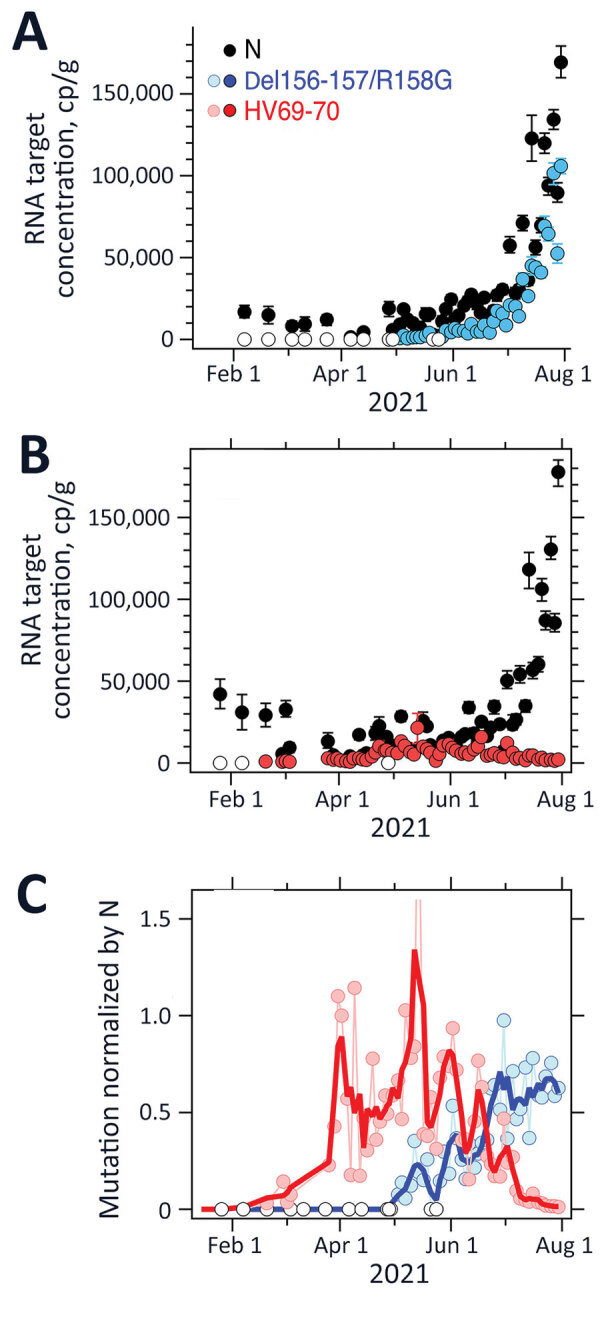
Measurements of severe acute respiratory syndrome coronavirus 2 variants of concern in wastewater solids, Sacramento, California, USA. Concentrations of N gene and mutations found in Delta (Del156-157/R158G; panel A), and Alpha (HV69-70; panel B) severe acute respiratory syndrome coronavirus 2 in wastewater solids and their ratio (panel C). Error bars in panels A and B represent SDs derived from the 10 replicates run for each sample; open white circles are nondetects ((below the limit of detection) and shown as 0. Errors include technical and replication errors. If error bars are not visible, then errors are smaller than the symbol. For Del156-157/R158G/N ratio, the smoothed line is a 3-point running average, and for the HV69-70/N ratio, the smoothed line is a 7-point running average; each approximates a weekly average. The timescale for the HV69-70 data (B) is truncated for visualization; additional data on dates before January 15, 2021, are described in the article and were nondetects. One data point is located beyond the upper bound of the y-axis (C): the value for HV69-70/N on May 14, 2021, was 2.4. N, nucleoprotein.

Results for positive and negative controls were as expected, and recovery controls indicated consistent RNA recovery from samples and lack of substantial inhibition (Appendix). We measured HV69-70 concentrations up to 1 time/day at San Jose and up to 3 times/week at Sacramento; concentrations ranged from not detected to >10,000 copies/g (Figure 1, 2). N and HV69-70 concentrations at San Jose before 15 Feb 2021 are not presented graphically; samples collected during July and September 2020 did not have measurable HV69-70. HV69-70 was measured for the first time in San Jose solids on November 25, 2020, at concentrations of ≈103 copy/g. We did not detect HV69-70 in Sacramento wastewater solids before late February 2021; results for samples collected in October 2020 (not shown in plot) and late January 2021 were nondetect for HV69-70. At both locations, the concentration of HV69-70 relative to the N gene (HV69-70/N ratio) increased over time beginning in early March 2021, peaked in early June 2021 at San Jose and May 2021 at Sacramento when HV69-70/N was ≈1, and then fell until HV69-70 became undetectable at San Jose and present at very low relative concentrations at Sacramento (0.01) in late July 2021 (Figure 1, 2).

Del156–157/R158G concentrations were measured as frequently as three times per week at both San Jose and Sacramento and ranged from not detected to 100,000 copies/g (Figure 1, 2). We observed Del156-157/R158G nondetects in samples collected before early April 2021 at both sites, and then both sites experienced a small peak in Del156–157/R158G concentration in early to mid-May 2021 (Del156–157/R158G relative to N ≈0.2–0.3 at the 2 sites), followed by a decline to undetectable levels over ≈2 weeks, followed by a sharp increase until the end of the data series. During this time, N gene concentrations in wastewater increased contemporaneously. The concentration of Del156–157/R158G relative to N (Del156–157/R158G/N ratio) (Figure 1, 2) increased to ≈0.8 at the sites by the end of the data series.

### Trends in Variants in Sequenced Case Isolates from Sewersheds

**Figure 3:**
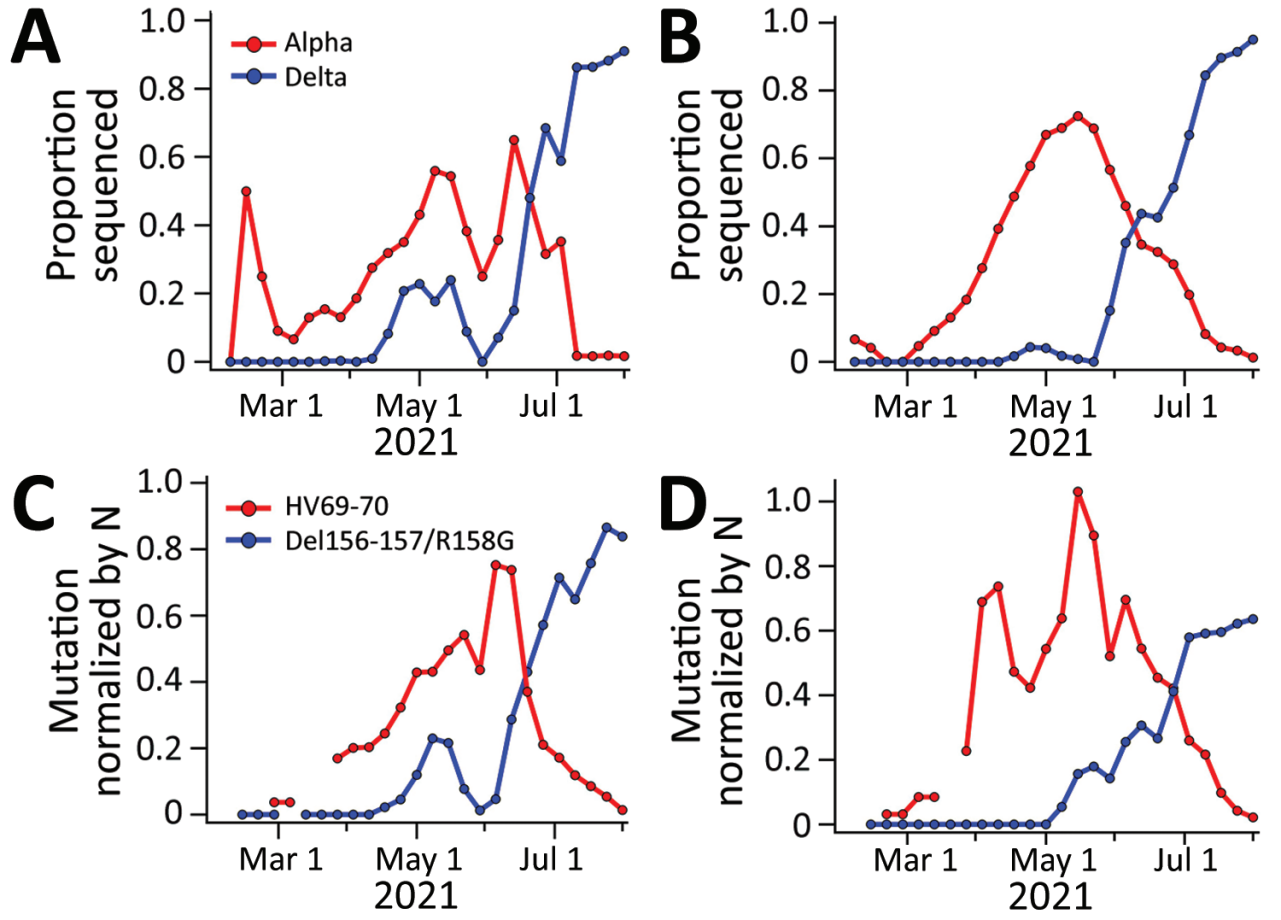
Comparison of severe acute respiratory syndrome coronavirus 2 variants of concern in wastewater solids with coronavirus disease case isolates, San Jose and Sacramento, California, USA, February 1–July 31, 2021. A, B) Proportion of circulating severe acute respiratory syndrome coronavirus 2 attributable to the Alpha and Delta variants, estimated from isolate sequencing data from cases collected and sequenced over the previous 14-day period in San Jose (A) and Sacramento (B) sewersheds. C, D) Concentrations of mutations found in Alpha (HV69-70) and Delta (Del156-157/R158G) variant viruses, normalized by N gene concentrations in wastewater, averaged over the previous 14 days in San Jose (C) and Sacramento (D). No data are shown for dates for which no measurements were made within the previous 14 days. N, nucleoprotein.

We analyzed trends in Alpha and Delta variants confirmed from case isolates collected from residents (case isolates) of the San Jose and Sacramento sewersheds from early February through late July 2021 (Figure 3). Alpha proportions increased in both sewersheds from early March, peaking in May–June and decreasing in early July. Delta was first identified in isolates in early April and by the end of July accounted for almost all sequenced isolates. In San Jose, a small peak in Delta occurred in May, before a large sustained increase in June; a similar peak is also evident, to a lesser extent, in the Sacramento Delta data. During this period, the 7-day average laboratory-confirmed incident COVID-19 cases ranged from 1 to 30/100,000 population (Appendix Figure 2) in each sewershed. Incident COVID-19 cases in each sewershed is positively and significantly correlated with N gene measurements in the settled solids (Pearson R [r_p_] 0.8, df 46–131, p<10^–10^ for both San Jose and Sacramento N gene datasets, regardless of whether they were generated when measuring the Delta or Alpha mutation).

### Relationship between Proportion of Alpha and Delta Variants in Case Isolates and Wastewater Mutation Data

We compared ratios of HV69-70 and Del156-157/R158G mutations to the N gene (VOC abundance estimates based on wastewater) against the proportion of all case isolates sequenced and identified as Alpha and Delta variant (VOC abundance estimates based on case isolate sequencing) from each sewershed (Figure 3). Trends of wastewater VOC abundance estimates follow closely and temporally the trends of case isolate sequencing VOC abundance estimates during this period at both sewersheds, including features such as an early peak in Delta in May. Alpha and Delta mutation gene ratios from wastewater were strongly correlated with the corresponding ratios of each VOC from case isolates sequenced: r_p_ 0.82 (p<10^–5^, df 19 in San Jose) and 0.88 (p<10^–7^, df 21 in Sacramento) for Alpha; r_p_ 0.97 (p<10^–15^, df 23 for both San Jose and Sacramento) for Delta. When compared to the opposing variant, the mutation gene ratios were not correlated (p>0.05 for all).

### Completeness of and Delays in Receiving SARS-CoV-2 Isolate Sequence Data

During February 1–August 1, 2021, the total number of case isolates sequenced over a 14-day period in our 2 sewersheds varied (2–520 median for 8% of all sequenced case isolates from San Jose and 6% from Sacramento). Earliest isolate sequencing results were available to CDPH ≈5 days after sample collection date. Approximately 75% of all sequenced isolate results in our dataset were available within 2–3 weeks. As more isolate sequencing data were received, estimated proportions of VOCs changed over time. Around 3 weeks were required for 95% of VOC estimates (14-day window) to be within 10% of the final estimate.

## Discussion

Our results show that the HV69-70 and Del156-157/R158G mutation assays as used for wastewater settled solids were sensitive and specific. By using these PCR mutation assays, we found strong correlation between wastewater estimates and case isolate sequencing–derived estimates of circulating Alpha and Delta in 2 large metropolitan communities in California, USA. Mutations were detected in wastewater samples collected 1–3 weeks earlier than when Alpha and Delta variant estimates generated by case-isolate sequencing were available and reliable. Targeted mutation assays applied to SARS-CoV-2 RNA extracted from wastewater solids can be a rapid, efficient, and reliable way to monitor VOCs introduced to and circulating in a community. Monitoring for VOCs using wastewater may provide earlier complementary surveillance data than from case isolate sequencing data, if mutation assays are or can be developed for new and existing VOCs and put into use in a timely manner.

Use of PCRs targeting characteristic mutations thought to be particular to a SARS-CoV-2 variant may concurrently detect other SARS-CoV-2 strains that carry the same mutations. Targeting a single mutation in wastewater, as was done in our study for Alpha, carries an increased potential risk for mischaracterization. For example, on September 8, 2021, according to GISAID (https://www.gisaid.org), a global repository of case isolate sequence data, 1,043,561 (97%) of the 1,077,360 Alpha (B.1.1.7 and Q sublineages) sequences contained the HV69-70 mutation. However, HV69-70 was also present in other variants, such as B.1.258.19, where it was present in all 141 B.1.258.19 sequences in GISAID, and B.1.617.2, where it was present in 647 (0.2%) of 402,038 sequences. Targeting multiple mutations, as was done in our study with Delta, can increase specificity. Of the 937,570 sequences in GISAID classified as Delta (B.1.617.2 and AY sublineages), 842,354 (90%) have the Del156–157/R158G mutations (referred to as E156G/del157–158 in GISAID). Although this combination of mutations can be present in other variants, it is rarer; the non-Delta variant with the highest percentage of sequences with these mutations is B.1.617.3, for which there were 266 isolates in the global GISAID database and only 77 (29%) possessing these mutations. The non-Delta variant with these mutations for which there are the largest number of isolates in GISAID is B.1.1.7, for which 6 (0.0006%) of the >1,053,637 million sequences have these mutations.

Our findings show that use of mutation assays (HV69-70 for Alpha, Del156-157/R158G for Delta) to estimate circulating variants in wastewater correlated well with estimates from case isolate sequencing data. Wastewater estimates for Alpha, based on a single deletion assay, were robust over time in 2 large municipalities over 8 months (r_p_ 0.82, p<10^–5^ in San Jose; r_p_ 0.88, p<10^–7^ in Sacramento), including periods of high (tail of 2020 winter, 2021 summer) and low (2021 spring) community SARS-CoV-2 transmission. Similarly, estimates for Delta, based on multiple mutations, correlated highly with estimates from sequenced case isolates (r_p_ 0.97, p<10^–15^ for both San Jose and Sacramento). Concurrent monitoring of VOCs in both wastewater and case isolates can confirm whether targeted mutation assays used are correlated with the VOCs being monitored and mitigate risks for misinterpreting wastewater results. Discrepant or divergent estimates between the 2 datasets should be noticeable within weeks and would suggest another variant with the same mutations circulating at abundance, prompting investigation if unexpected.

Emergence of the Omicron VOC in November 2021 (*3*) provides an excellent example of the importance of interpreting wastewater mutation assay data in the context of case isolate sequencing data. Omicron also includes the HV69-70 mutation. At the time this study was conducted, the HV69-70 mutation, as noted previously, was rarely circulating in non-Alpha variants, suggesting that positive detections likely represented Alpha. However, Alpha disappeared from California circulation by end of summer 2021 and by December 2021, public health concern was for Omicron. With zero Alpha case isolates detected in either sewershed during September 1–December 1, the HV69-70 mutation assay was deployed on wastewater to screen for presence of Omicron, a more likely VOC to emerge in California than Alpha, while a more specific assay was developed (*22*).

For validated assays deployed in established wastewater sites, wastewater surveillance for VOCs could be an important adjunctive estimate of variant circulation. Because cost and limited genomic testing capacity make sequencing all COVID-19 isolates impractical, especially during times of high case incidence, health departments and decision-makers extrapolate information from relatively small numbers or proportions of sequenced isolates, which may be biased and unrepresentative. For our case dataset, 14-day VOC estimates were derived from as few as 2–20 total case isolates and <1% of total cases sequenced.

Wastewater variant monitoring can overcome biases and delays seen with case isolate sequencing. Because everyone living in a sewershed contributes waste to the system, wastewater monitoring is independent of testing and care accessibility biases and results are more representative of cases in that sewershed. In addition, wastewater mutation assay results are available in a shorter time than VOC estimates from sequencing of case isolates. In our monitored sewersheds, the total average turnaround time from wastewater collection to testing results was <8 hours. In contrast, for our 2 sewersheds, it took 2–3 weeks after sample collection date for 75% of case isolate sequence results to be received and 3 weeks for most 14-day VOC estimates to be within 10% of their final estimate. These delays do not include the additional delay between case symptom onset and test taking that could further accentuate time advantages of wastewater variant monitoring.

Several limitations exist for using wastewater (solids or liquids) for SARS-CoV-2 variant monitoring. Laboratory limits of detection for SARS-CoV-2 RNA in wastewater and for targeted mutations may result in no detection, especially at times with lower community COVID-19 case counts and consequent lower overall concentrations of SARS-COV-2 RNA in wastewater. However, even in mid-May 2021, when case counts in these 2 sewersheds were as low as 1–2 cases/100,000 population, both SARS-CoV-2 RNA levels and variant abundance could still be measured and accurately estimated. Estimates of circulating Alpha and Delta mutations were also able to be consistently detected even at levels <5% of total SARS-CoV-2 RNA. Limits of detection, both for SARS-CoV-2 and for different mutations associated with variants, are likely to vary depending on laboratory methods used and which mutation is targeted; delineating these limits for each laboratory, sewershed, and assay is important for interpreting what a nondetect result implies about variant circulation.

Because newly identified variants to be monitored require new mutation assays to be designed, the time needed to design, test, and begin using assays is a crucial consideration (Appendix Figure 3). Although the time to design an assay in silico (<1 day) and test its sensitivity and specificity in vitro (3–5 days) is short, the time to receive reagents, including synthesized oligos and positive control RNA, from vendors can take 4–6 weeks because of supply chain issues and increased demand during the pandemic. In addition, before an assay can be designed, variant sequences and mutations must be accurately characterized, which can delay the assay design process. Efforts to develop assays before variants become VOCs and proactively order reagents can help ensure assays are available when needed for public health response.

Monitoring for VOCs will continue to be an important public health function and a need that will become more salient if SARS-CoV-2 testing of cases and sequencing resources or utilization decrease over time. Difficulty in surveillance based on case isolate sequencing, including difficulties attributable to nonrepresentative sampling and delayed results, mean that complementary variant surveillance methods are needed. Detection and monitoring of variants in wastewater has been proposed as an adjunct methodology, and our experiences monitoring for 2 VOCs in 2 large California municipalities support the use of targeted PCR mutation assays as a useful method to estimate abundance of circulating VOCs and inform public health. In conjunction with continued COVID-19 case isolate sequencing, wastewater variant monitoring can be strategically deployed as an adjunct public health surveillance tool.

Appendix Additional information about estimating relative abundance of 2 SARS-CoV-2 variants through wastewater surveillance at 2 large metropolitan sites, United States.
