# Supplementary material for: Estimating Relative Abundance of 2 SARS-CoV-2 Variants through Wastewater Surveillance at 2 Large Metropolitan Sites, United States
Source: Emerg Infect Dis. 2022 May;28(5):940–7. doi: 10.3201/eid2805.212488 (PMC9045426; doi:10.3201/eid2805.212488)
Supplement: Appendix — Additional information about estimating relative abundance of 2 SARS-CoV-2 variants through wastewater surveillance at 2 large metropolitan sites, United States. [file 21-2488-Techapp-s1.pdf]

Article DOI: https://doi.org/10.3201/eid2805.212488

Estimating Relative Abundance of 2 SARS-CoV-2 Variants through Wastewater Surveillance at 2 Large Metropolitan Sites, United States

Appendix

Supplementary Methods

Sampling information

Samples were collected by POTW staff using sterile technique in clean, labeled bottles. Approximately 50 ml of settled solids were collected each study day from each POTW. Settled solids samples were grab samples at SAC. It should be noted that "grab" samples from the sludge line of the primary clarifier represent composites of solids settled over the residence time of the clarifier. At SJ, POTW staff manually collected a 24 h composite sample. Samples were immediately stored at 4°C and transported to the lab and processed (within 6 hours of collection).

Mutation assay design

Assays were developed in silico using Primer3Plus (https://primer3plus.com/) to target mutations present in Alpha and Delta variants of concern. Mutation sequences were obtained from outbreak.info/compare-lineages. The parameters used in the development are provided in the list following this paragraph. Primers and probe sequences are provided in Appendix Table and schematic of the assays are provided in Appendix Figure 4 and Appendix Figure 5.

Parameters used with primer design software.

- Product size ranges: 60–200

- Primer size: min 15, opt 20, max 36

- Primer melting temperature: min 50°C, optimal 60°C, max 65°C

- GC% content: min 40%, optimal 50%, high 60%
- concentration of divalent cations = 3.8 mM
- concentration of dNTPs needs to be 0.8 mM
- Internal Oligo: size min 15, optimal 20, max 27
- Internal Oligo: Melting temp min 62°C, optimal 63°C, max 70°C
- Internal Oligo: GC% min 30%, optimal 50%, max 80%

Specificity Screening Against Other Targets

Primers and probe sequences were screened for specificity tested in vitro against a respiratory virus panel (NATtrol Respiratory Verification Panel, Zeptomatrix) that includes several influenza and coronavirus viruses, "wild-type" gRNA from SARS-CoV-2 strain 2019-nCoV/USA-WA1/2020 (ATCC® VR-1986D) which does not contain the mutations (hereafter referred to as WT-gRNA), heat inactivated SARS-CoV-2 strain Alpha (SARS-CoV-2 variant B.1.1.7, ATCC® VR-3326HK), and synthetic gRNA from Twist Biosciences (South San Francisco, California, USA) for Beta (Twist control 16), Gamma (Twist control 17), Kappa (Twist control 18), and Delta (Twist control 23) variants. RNA was extracted from the virus panel and whole viruses using the Chemagic Viral DNA/RNA 300 Kit H96 for the Perkin Elmer Chemagic 360. RNA was used undiluted as template using digital droplet RT-PCR with mutation primer and probes in singleton assays in single wells (see further details on digital PCR below). The concentration of targets used in the in vitro specificity testing was ≈275 copies per well.

Sensitivity and Specificity

For sensitivity and specificity testing, single wells were run using the HV69–70 assay for a subset of no and low background samples, but otherwise run in three wells. The number of copies of variant mutation sequences input to each well was estimated using a dilution series of variant gRNA in no background; the vendor specified concentration of the variant gDNA or gene blocks was scaled by the slope of the curve relating the measured ddRT-PCR concentration and the calculated input concentration based on the vendor estimates. Additional details of these experiments are provided in the main text of the paper.

Solids Sample Processing

The solids were dewatered by centrifugation at 24,000 x g for 30 minutes at 4°C. The supernatant was aspirated and discarded. A 0.5 - 1 g aliquot of the dewatered solids was dried at 110°C for 19–24 hrs to determine its dry weight. Bovine coronavirus (BCoV) was used as a positive recovery control. Each day, attenuated bovine coronavirus vaccine (PBS Animal Health, Calf-Guard Cattle Vaccine) was spiked into DNA/RNA shield solution (Zymo Research) at a concentration of 1.5 µL /mL. Dewatered solids were resuspended in the BCoV-spiked DNA/RNA shield to a concentration of 75 mg/mL. This concentration of solids was chosen as previous work titrated solutions with varying concentrations of solids to identify a concentration at which inhibition of the SARS-CoV-2 assays was minimized (*5*). Between five and ten 5/32" Stainless Steel Grinding Balls (OPS Diagnostics) were added to each sample which was subsequently homogenized by shaking with a Geno/Grinder 2010 (Spex SamplePrep). Samples were then briefly centrifuged to remove air bubbles introduced during the homogenization process, and then vortexed to re-mix the sample.

RNA was extracted from 10 replicate aliquots per sample. For each replicate, RNA was extracted from 300 µl of solids sample using the Chemagic Viral DNA/RNA 300 Kit H96 for the Perkin Elmer Chemagic 360 followed by PCR Inhibitor Removal with the Zymo OneStep-96 PCR Inhibitor Removal Kit. Extraction negative controls (water) and extraction positive controls were extracted using the same protocol as the solids samples. The positive controls consisted of 500 copies of WT-gRNA in the BCoV-spiked DNA/RNA shield solution described above. Undiluted extract was used for the SARS-CoV-2 assay template and a 1:100 dilution of the extract was used for the BCoV / PMMoV assay template. PMMoV is pepper mild mottle virus and is found in high abundance in feces and wastewater globally and is used here as a fecal strength control and an internal recovery control.

ddRT-PCR

Digital droplet RT-PCR assays for the mutations and N gene targets (see Appendix Table for primer and probe sequences, purchased from IDT) were performed on 20 µl samples from a 22 µl reaction volume, prepared using 5.5 µl template, mixed with 5.5 µl of One-Step RT-ddPCR Advanced Kit for Probes (Bio-Rad 1863021), 2.2 µl Reverse transcription, 1.1 µl DTT and primers and probes at a final concentration of 900 nM and 250 nM respectively. Droplets were generated using the AutoDG Automated Droplet Generator (Bio-Rad). PCR was performed

using Mastercycler Pro with cycling conditions described below. Droplets were analyzed using the QX200 Droplet Reader (Bio-Rad). All liquid transfers were performed using the Agilent Bravo (Agilent Technologies).

PCR was performed using Mastercycler Pro with the following cycling conditions: reverse transcription at 50°C for 60 minutes, enzyme activation at 95°C for 5 minutes, 40 cycles of denaturation at 95°C for 30 seconds and annealing and extension at either 59°C (for single or multiplex SARS-CoV-2 assays that include HV69–70, or do not include any mutation), 61°C (for single or multiplex assays that include Del156–157/R158G) or 56°C (for PMMoV/BCoV duplex assay) for 30 seconds, enzyme deactivation at 98°C for 10 minutes then an indefinite hold at 4°C. The ramp rate for temperature changes were set to 2°C/second and the final hold at 4°C was performed for a minimum of 30 minutes to allow the droplets to stabilize.

For the assay sensitivity and specificity testing, assays were run in single wells, or triplicate wells as specified above; each plate contained positive and negative PCR controls. Each wastewater sample was run in 10 replicate wells, and each plate of wastewater samples included extraction negative controls that were run in 7 wells, and extraction positive controls in 1 well. In addition, PCR positive controls for each target assayed on the plate were run in 1 well, and NTC were run in 7 wells.

Positive controls consisted of BCoV and PMMoV gene block controls (dsDNA purchased from IDT), gRNA of SARS-CoV-2 (strain 2019-nCoV/USA-WA1/2020, ATCC® VR-1986D), gRNA of SARS-CoV-2 Alpha lineage (SARS-CoV-2 variant B.1.1.7, ATCC® VR-3326HK), and gene block controls for Del156–157/R158G. Results from replicate wells were merged for analysis. Thresholding was done using QuantaSoft Analysis Pro Software (Bio-Rad, version 1.0.596). In order for a sample to be recorded as positive, it had to have at least 3 positive droplets. For the wastewater samples, three positive droplets corresponds to a concentration between ~500–1000 cp/g; the range in values is a result of the range in the equivalent mass of dry solids added to the wells.

For the wastewater samples, concentrations of RNA targets were converted to concentrations per dry weight of solids in units of copies/g dry weight using dimensional analysis. The total error is reported as standard deviations and includes the errors associated with the Poisson distribution and the variability among the 10 replicates. The recovery of BCoV was

determined by normalizing the concentration of BCoV by the expected concentration given the value measured in the spiked DNA/RNA shield. BCoV was used solely as a process control; samples were rerun in cases where the recovery of BCoV was less than 10%. PMMoV concentrations at the POTW were consistent within POTWs (Appendix Figure 6) and within the ranges observed previously suggesting consistent recovery of RNA across samples. All wastewater data, along with additional reporting details per the MIQE (*5*) and EMMI (*6*) guidelines are available publicly at the Stanford Digital Repository (https://doi.org/10.25740/zf117dn1545). The detection limits for the endogenous wastewater RNA targets are as follows: 500–1000 copies/g dry weight for all SARS-CoV-2 RNA gene targets, and 5×10^5–10×10^5 copies/g for the PMMoV gene target. The range varies from sample-to-sample and depends on the wet weight of the solids suspended in the DNA/RNA shield.

Effect of Storage on RNA

RNA samples from San Jose (SJ) were stored between 0 and 7 days before they were analyzed for HV69–70 and the N gene in a multiplex assay. SJ and Sacramento RNA samples were stored between 15 and 190 days before analysis for Del156–157/R158G and the N gene; Sacramento RNA samples were stored between 15 and 300 days before being analyzed for HV69–70 and the N gene. The N gene was run a second time in all samples as a check for RNA degradation during storage.

Additional Details on Incident COVID-19 Cases and COVID-19 Case Isolate Sequences

Sewershed service area boundary shapefiles were provided by each POTW and compared to postal code boundaries (2020 U.S. Census TIGER/Line shapefiles) in ArcGIS Pro version 2.7.3 (ESRI, Redlands CA). All postal codes with >50% of land area within sewershed boundaries were considered within the POTW service area and used for further analysis.

Supplementary Results

Recovery of BCoV was higher than 10% in all samples suggesting acceptable viral RNA recovery, and absence of substantial inhibition. PMMoV concentrations ranged from 108 to 109 copies per gram at both plants (Appendix Figure 6) on the sampling dates included in this study, within expected ranges previously measured at the POTWs (*7*) and indicating consistent

recovery of viral RNA from the wastewater solids. Negative controls including extraction and PCR negative controls were negative on all plates and positive controls all amplified. N genes concentrations measured contemporaneously with the mutation targets were similar to those measured immediately after wastewater sample collection (average ratio of the measurements ~ 0.9) indicating RNA stability during −80°C storage.

References

1. Haramoto E, Kitajima M, Kishida N, Konno Y, Katayama H, Asami M, et al. Occurrence of pepper mild mottle virus in drinking water sources in Japan. Appl Environ Microbiol. 2013;79:7413–8. PubMed https://doi.org/10.1128/AEM.02354-13

2. Zhang T, Breitbart M, Lee WH, Run J-Q, Wei CL, Soh SWL, et al. RNA viral community in human feces: prevalence of plant pathogenic viruses. PLoS Biol. 2006;4:e3. PubMed https://doi.org/10.1371/journal.pbio.0040003

3. Decaro N, Elia G, Campolo M, Desario C, Mari V, Radogna A, et al. Detection of bovine coronavirus using a TaqMan-based real-time RT-PCR assay. J Virol Methods. 2008;151:167–71. PubMed https://doi.org/10.1016/j.jviromet.2008.05.016

4. Wolfe MK, Topol A, Knudson A, Simpson A, White B, Vugia DJ, et al. High-frequency, high-throughput quantification of SARS-CoV-2 RNA in wastewater settled solids at eight publicly owned treatment works in northern California shows strong association with COVID-19 incidence. mSystems. 2021;6:e00829–21. PubMed https://doi.org/10.1128/mSystems.00829-21

5. Huggett JF; dMIQE Group. The Digital MIQE guidelines update: minimum information for publication of quantitative digital PCR experiments for 2020. Clin Chem. 2020;66:1012–29. PubMed https://doi.org/10.1093/clinchem/hvaa125

6. Borchardt MA, Boehm AB, Salit M, Spencer SK, Wigginton KR, Noble RT. The environmental microbiology minimum information (EMMI) guidelines: qPCR and dPCR quality and reporting for environmental microbiology. Environ Sci Technol. 2021;55:10210–23. PubMed https://doi.org/10.1021/acs.est.1c01767

7. Wolfe MK, Archana A, Catoe D, Coffman MM, Dorevich S, Graham KE, et al. Scaling of SARS-CoV-2 RNA in settled solids from multiple wastewater treatment plants to compare incidence rates of laboratory-confirmed COVID-19 in their sewersheds. Environ Sci Technol Lett. 2021;8(5):393–404. https://doi.org/10.1021/acs.estlett.1c00184

Appendix Table. Primer and probe sequences*

Target	Primer/Probe	Sequence
N Gene	Forward	CATTACGTTTGGTGGACCCT
	Reverse	CCTTGCCATGTTGAGTGAGA
	Probe	CGCGATCAAAACAACGTCGG (5′ FAM/ZEN/3′ IBFQ)
BCoV	Forward	CTGGAAGTTGGTGGAGTT
	Reverse	ATTATCGGCCTAACATACATC
	Probe	CCTTCATATCTATACACATCAAGTTGTT (5′ FAM/ZEN/3′ IBFQ)
PMMoV	Forward	GAGTGGTTTGACCTTAACGTTTGA
	Reverse	TTGTCGGTTGCAATGCAAGT
	Probe	CCTACCGAAGCAAATG (5′ HEX/ZEN/3′ IBFQ)
HV69–70 del (Alpha)	Forward	ACTCAGGACTTGTTCTTACCT
	Reverse	TGGTAGGACAGGGTTATCAAAC
	Probe	ATGCTATCTCTGGGACCAAT (5′ FAM or HEX/ZEN/3′ IBFQ)
Del156–157/R158G (Delta)	Forward	ATTCGAAGACCCAGTCCCTA
	Reverse	AGGTCCATAAGAAAAGGCTGA
	Probe	TGGATGGAAAGTGGAGTTTATTCTAG (5′ FAM or HEX/ZEN/3′ IBFQ)

*The N gene assay is described in detail in Huisman et al. (J.S. Huisman et al., unpub. data, https://doi.org/10.1101/2021.04.29.21255961). The PMMoV assay is from Haramoto et al. (*5*) and Breitbart et al. (*6*). The BCoV assay is from Decaro et al. (*7*).

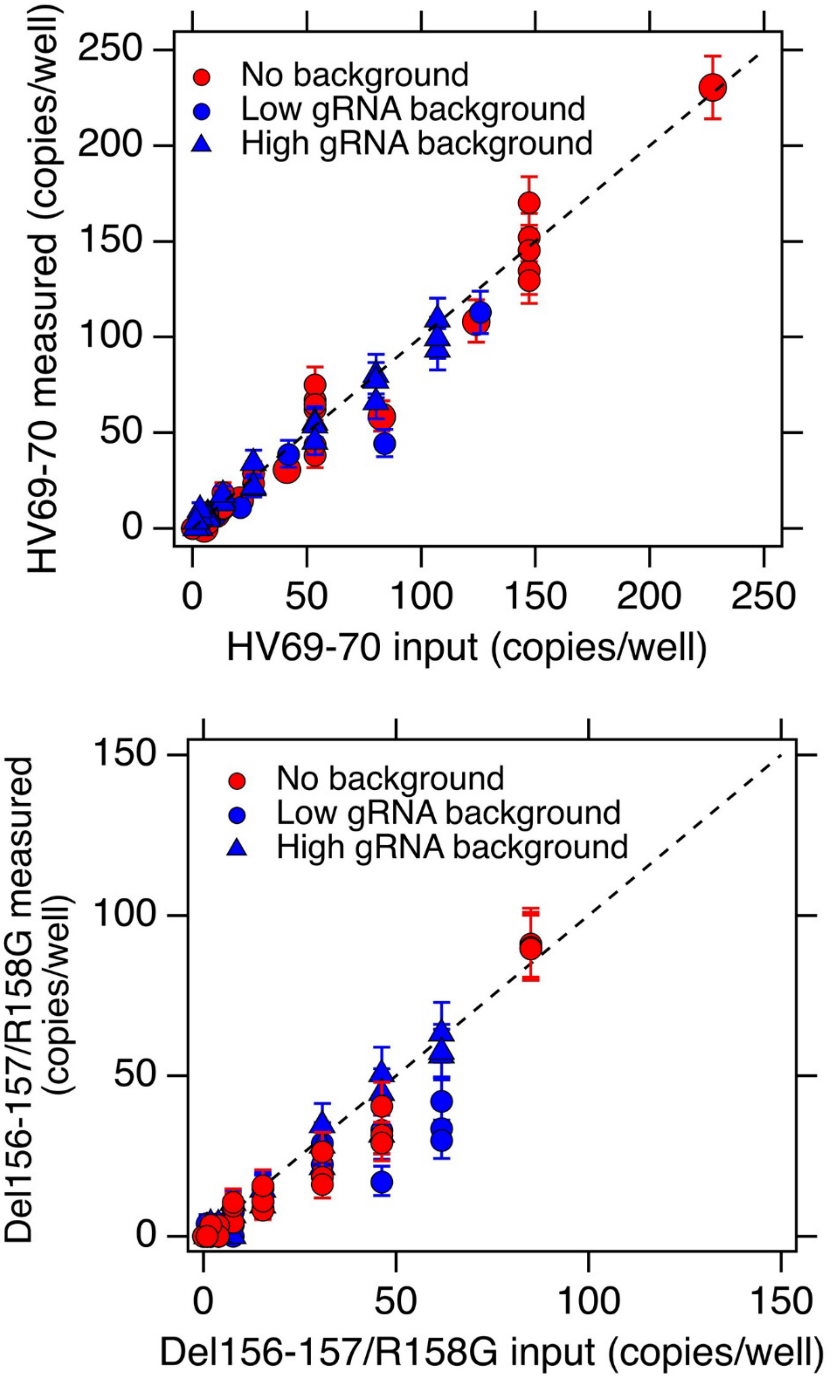

Appendix Figure 1. Measured copies of mutation targets versus input copies of mutation targets in no, low, (100 copies/well) and high (10,000 copies/well) background of WT-gRNA from 2019-nCoV/USA-WA1/2020 which does not contain the mutations. Error bars represent standard deviations of the measurements. No background is shown in red; and measurements where background gRNA was included are shown in blue. We were able to detect concentrations as low as 5 copies/well for the mutation assays when there was no, low, and high background of WT-gRNA.

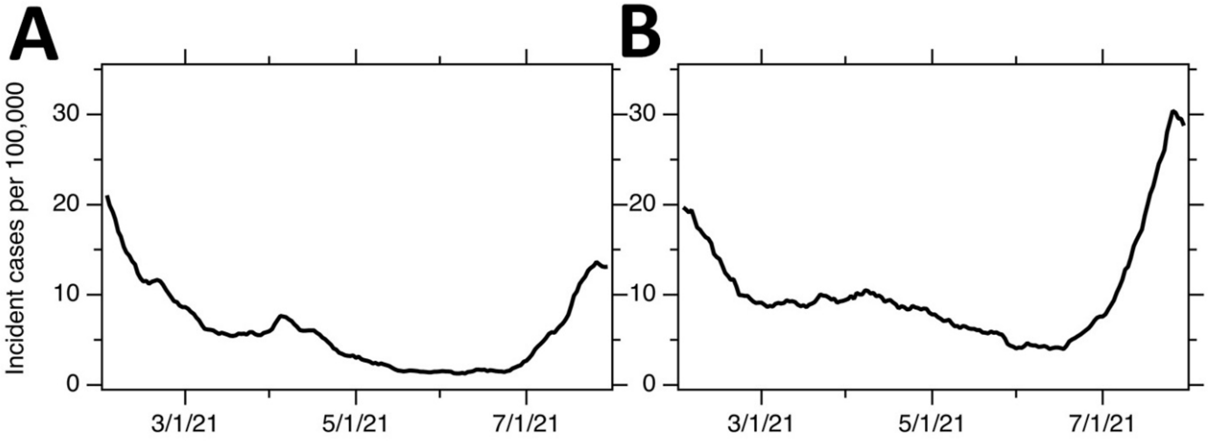

Appendix Figure 2. Incident cases of COVID-19 (seven day smoothed) in the sewersheds.

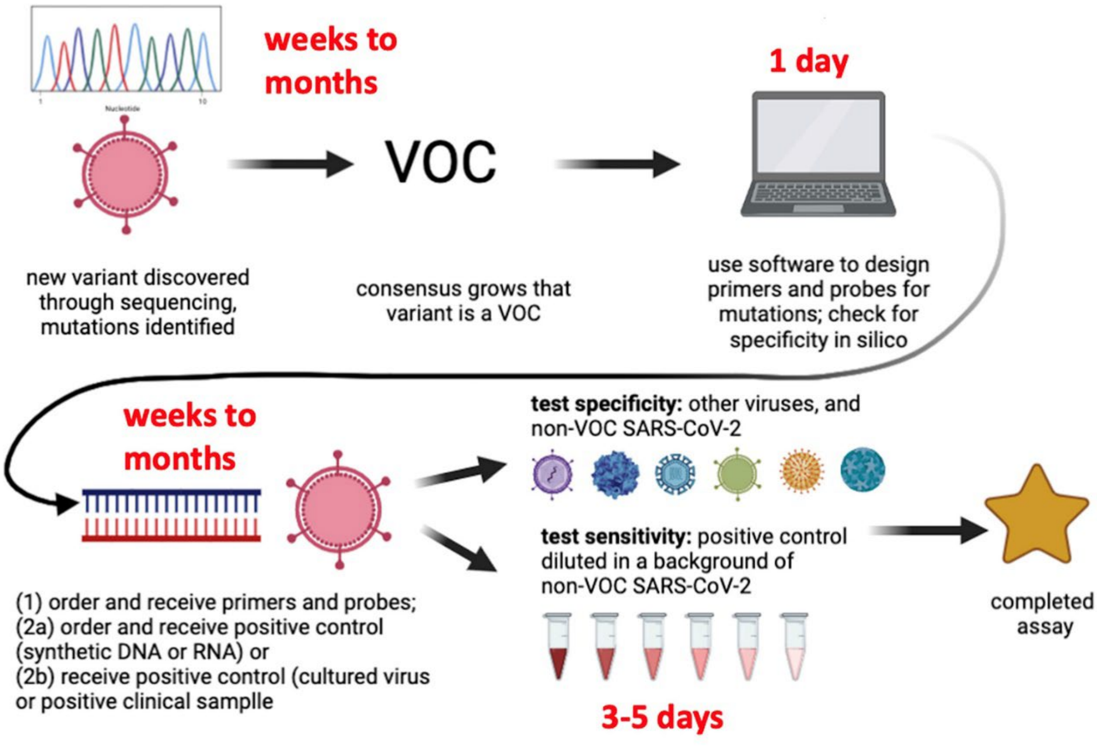

Appendix Figure 3. Timeline to develop completed variant mutation assay. The time for variant discovery to consensus that variant is a VOC and time for receiving reagents are obstacles for the rapid development of mutation assays. Figure was created using Biorender.com.

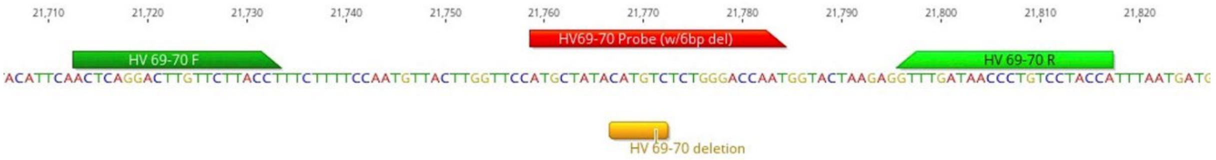

Appendix Figure 4. Location of HV69–70 primers and probes within the SARS-CoV-2 genome. Probe was designed with the deletion in the center (image shows the location, but the annotated probe has a 6bp deletion at amino acids 69 and 70).

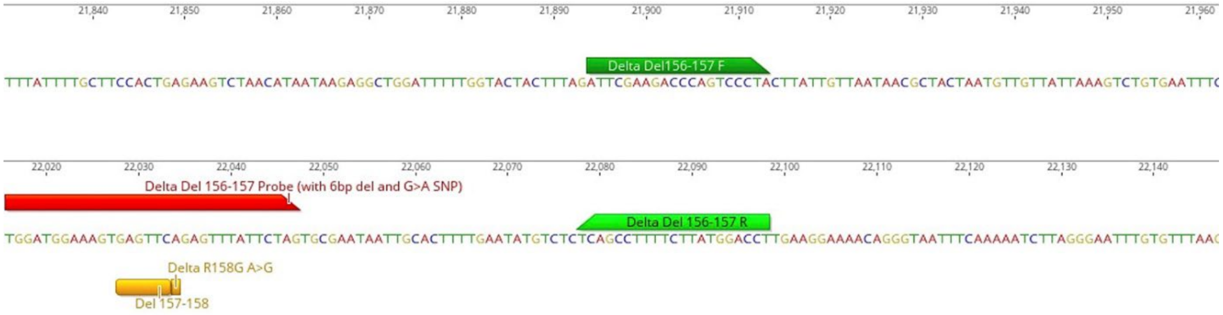

Appendix Figure 5. Location of Del156–157/R158G primers and probes within the SARS-CoV-2 genome. Probe was designed with the deletion in the center.

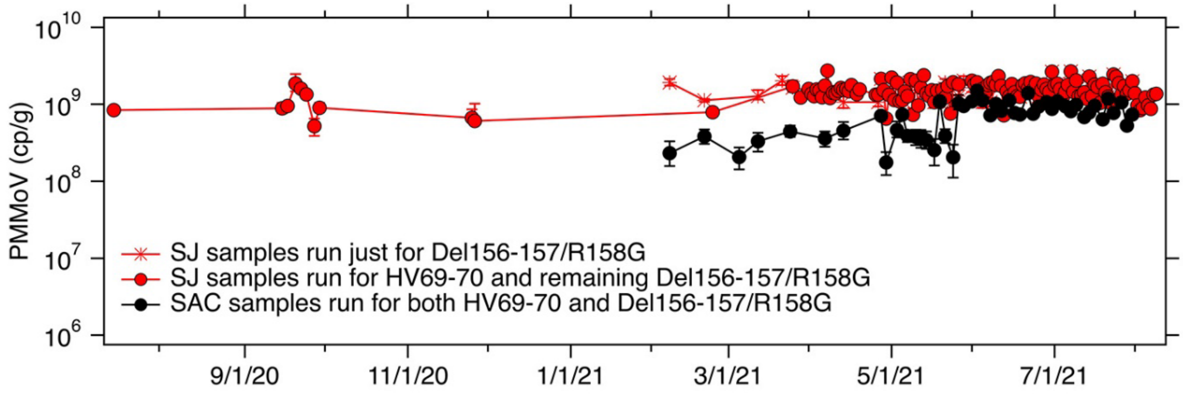

Appendix Figure 6. PMMoV RNA target concentrations in wastewater solids used in this study in units of copies per gram dry weight. PMMoV is used as an endogenous control as we expect it in high concentrations and low concentrations would suggest failure in extraction efficiency.
